# Liquid biopsy in gastrointestinal oncology: clinical applications and translational integration of ctDNA, CTCs, and sEVs

**DOI:** 10.3389/or.2025.1702932

**Published:** 2025-10-20

**Authors:** Rita Palieri, Maria De Luca, Francesco Balestra, Giorgia Panzetta, Claudio Lotesoriere, Federica Rizzi, Angela Dalia Ricci, Rita Mastrogiacomo, Maria Lucia Curri, Luigi Andrea Laghi, Gianluigi Giannelli, Nicoletta Depalo, Maria Principia Scavo

**Affiliations:** ^1^ Laboratory of Molecular Medicine, National Institute of Gastroenterology IRCCS “S. de Bellis”, Bari, Italy; ^2^ Medical Oncology Unit, National Institute of Gastroenterology, IRCCS “S. de Bellis” Research Hospital, Castellana Grotte, Italy; ^3^ Institute for Chemical-Physical Processes, Italian National Research Council (IPCF)-CNR SS Bari, Bari, Italy; ^4^ National Interuniversity Consortium of Materials Science and Technology (INSTM), Bari Research Unit, Bari, Italy; ^5^ Department of Chemistry, University of Bari, Bari, Italy; ^6^ Department of Medicine and Surgery, University of Parma, Parma, Italy; ^7^ Scientific Direction, National Institute of Gastroenterology IRCCS “S. de Bellis”, Bari, Italy

**Keywords:** liquid biopsy, gastrointestinal cancer (GI), circulating tumor cells (CTC), extracellular vesicles (EVs), circulating tumor DNA (ctDNA)

## Abstract

**Background and aims:**

Liquid biopsy offers a minimally invasive tool to detect actionable mutations, monitor minimal residual disease (MRD), and guide therapy in gastrointestinal (GI) cancers. We critically review the clinical utility of circulating tumor DNA (ctDNA), circulating tumor cells (CTCs), and small extracellular vesicles (sEVs) across GI malignancies and propose a framework for their integration into clinical practice.

**Methods:**

We synthesized evidence from over 200 studies, including prospective trials and translational research, to assess diagnostic accuracy, prognostic value, and clinical actionability of each biomarker type in esophageal, gastric, colorectal, pancreatic, hepatocellular, and biliary cancers.

**Results:**

ctDNA has shown strong potential for MRD detection and treatment monitoring, particularly in colorectal and pancreatic cancer. CTCs offer insights into metastatic risk and therapeutic resistance, while sEVs provide molecular cargo relevant to immunomodulation and disease progression. Emerging microfluidics and AI-driven multi-omics approaches may overcome current limitations.

**Conclusion:**

The integration of liquid biopsy technologies into GI oncology holds promise for early detection and precision therapy. We propose a five-phase clinical roadmap and outine the key research gaps that need to be addressed before widespread implementation in routine care.

## Graphical Abstract

**Figure:**
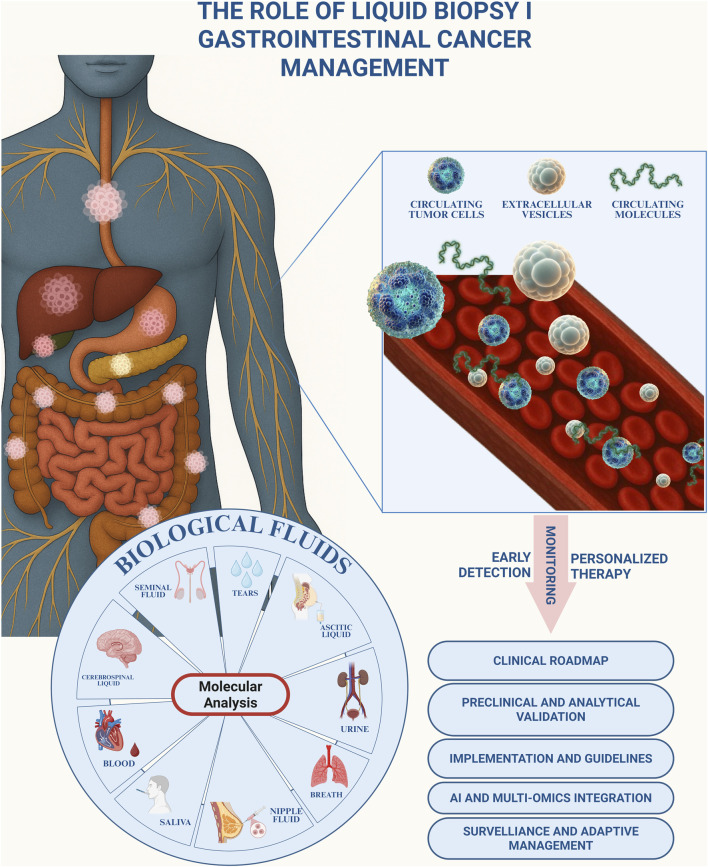
Graphical abstract illustrating the role of liquid biopsy in the management of gastrointestinal cancer. Tumor-derived biomarkers, including circulating tumor cells, extracellular vesicles, and circulating molecules, can be detected in different biological fluids (blood, saliva, urine, cerebrospinal fluid, seminal fluid, nipple fluid, ascitic liquid, tears, and breath) through molecular analysis. Liquid biopsy enables early detection, longitudinal monitoring, and the development of personalized therapeutic strategies. Key translational aspects include the establishment of a clinical roadmap, preclinical and analytical validation, implementation of standardized guidelines, integration of artificial intelligence and multi-omics approaches, as well as surveillance and adaptive management.

## 1 Introduction

**FIGURE 1:**
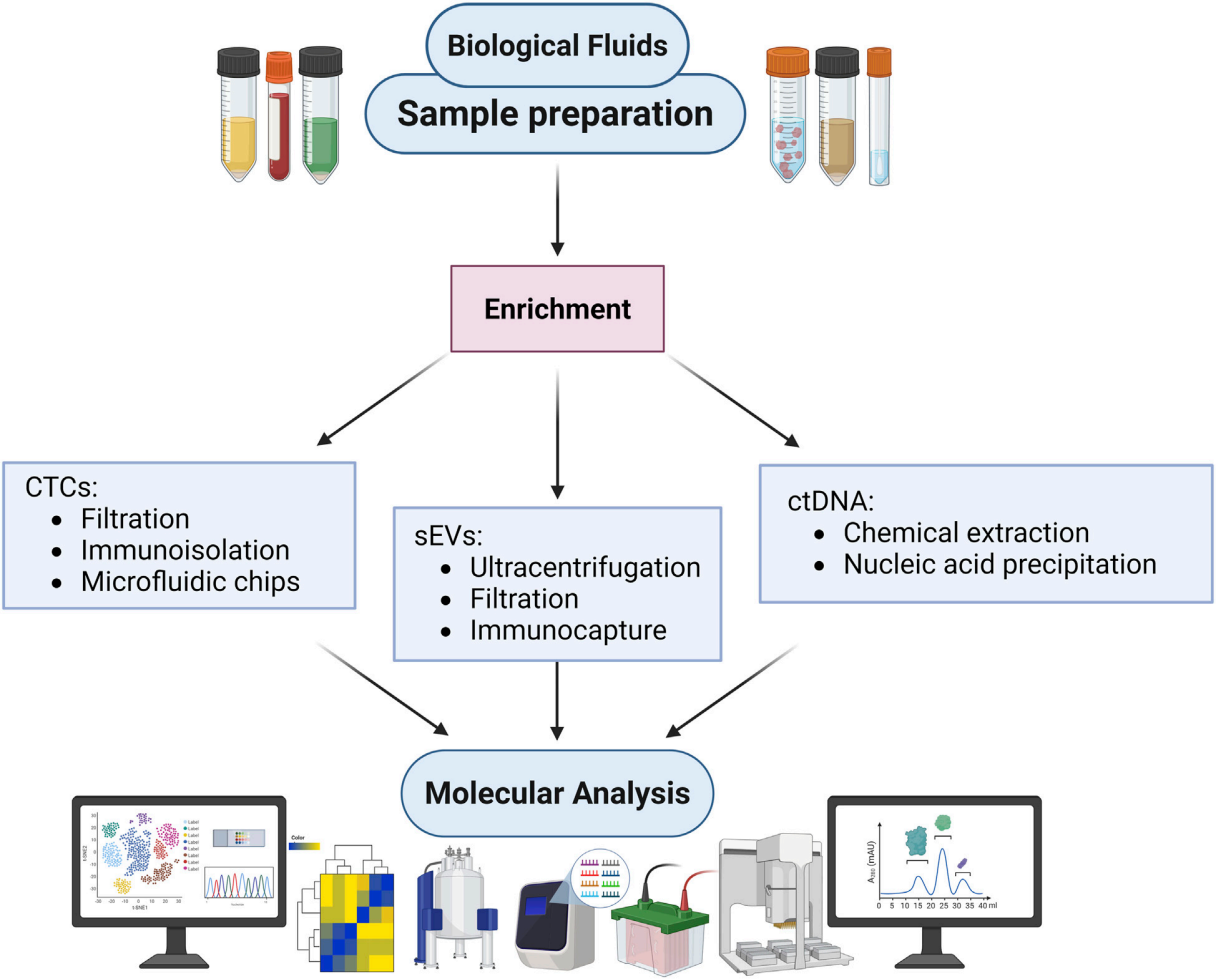
Fraction of Liquid Biopsy derived from body fluids. Overview of biological fluids utilized for diagnostic and research purposes. The outer ring identifies different types of biological fluids, including seminal fluid, tears, ascitic liquid, urine, breath, nipple fluid, saliva, blood, and cerebrospinal fluid. The inner section highlights key circulating components present within these fluids, such as circulating tumour cells (CTCs), circulating molecules (e.g., DNA, RNA, and proteins), and small extracellular vesicles (sEVs), that are obtained from liquid biopsy.

Cancer is the world’s second-deadliest disease, making early detection vital. While tissue biopsy is still the diagnostic gold standard, it is invasive and often misses tumour diversity or changes over time. Liquid biopsy, by analysing tumour-derived material in blood, saliva, urine, or other fluids, provides a non-invasive, real-time, and more comprehensive picture of tumour biology and progression (1). This innovative diagnostic method minimizes patient discomfort and enables real-time monitoring of tumour evolution and therapeutic responses (Figure 1) (2). Additionally, tissue biopsies may be unsuitable for detecting tumours at early stages (3). In gastrointestinal (GI) cancers, often marked by late diagnoses and limited treatment options, liquid biopsy offers a more precise approach to disease management (4). Key biomarkers include circulating tumour cells (CTCs), extracellular vesicles (EVs), and circulating tumour DNA (ctDNA). CTCs are cancer cells shed into the bloodstream from primary or metastatic sites (5), and their detection often relies on epithelial markers (e.g., EpCAM, Cytokeratin) or size and density differences. Advances in single cell sequencing of CTCs provide valuable insights into genetic heterogeneity and resistance mechanisms (6). EVs constitute a diverse population of membrane-bound vesicles secreted by most cell types and found in biological fluids. Small EVs (sEVs, <200 nm), among them exosomes, are the most extensively studied subclass due to their involvement in both physiological and pathological processes. They play key roles in intercellular communication by transferring bioactive molecules, and increasingly studied for their involvement in disease pathogenesis, diagnostics, and therapeutics (7). In liquid biopsy, sEVs have gained prominence due to their ability to carry proteins, lipids, nucleic acids (DNA, mRNA, non-coding RNA), and metabolites. These molecular cargos reflect the physiological and pathological states of their originating cells, making sEVs valuable biomarkers. They hold significant potential for early cancer detection, prognostic assessment, and therapeutic monitoring, providing insights into tumour biology and aiding in personalized oncology strategies (8). In GI tumourigenesis, sEVs promote cancer progression by remodelling the microenvironment, enhancing angiogenesis, and modulating immune responses, supporting metastasis (9–12). Their non-invasive detection in body fluids enables the monitoring of disease progression, therapeutic responses, and recurrence. sEVs-based assays improve diagnostic accuracy, patient stratification, and clinical decision-making in GI cancers (13). Similarly, ctDNA is an important component of liquid biopsy approaches, consisting of short nucleic acid fragments released into the bloodstream by cancer cells through apoptosis, necrosis, or active secretion (14). ctDNA mirrors the genetic and epigenetic landscape of its tumour, enabling non-invasive liquid biopsy for GI cancers. It supports early detection, surveillance of progression, minimal residual disease (MRD), recurrence, and treatment response. In colorectal, gastric, and pancreatic cancers, ctDNA detects mutations, resistance, and relapse risk, guiding personalized therapy (15–17). This review aims to examine the advances in liquid biopsy technologies and critically assess their significant clinical potential for each type of most common GI cancer. It explores recent developments in these technologies and evaluates their impact on the clinical management of GI cancers (Figure 1).

## 2 Technological landscape and pre-analytical considerations

Fragile circulating biomarkers demand ultrasensitive workflows, combining next-generation sequencing with error-suppression barcodes, digital PCR and droplet digital PCR, each paired with purpose-built enrichment modalities (18). CTC pre-enrichment, microfluidic capture of sEVs and quantitative ctDNA assays now sharpen MRD detection, serial therapeutic monitoring and immediate, data-driven treatment adjustment (19–21). Whole blood must be drawn into stabilising tubes, transported promptly and processed under cold-chain control to preserve biomolecule integrity from deviations accelerate degradation. Pre-analytical disparities collection tubes, centrifugation speeds, storage times and heterogeneous sequencing or PCR platforms foster pronounced inter-laboratory variability, complicating meta-analysis and reproducible standardisation efforts (22, 23). Enrichment exploits physical and biological differences: size exclusion filters eliminate smaller haematologic cells, while immunoisolation seizes tumour cells via EpCAM or other surface markers. Cutting edge microfluidic chips integrate size selective and antigen specific traps within nanoscale channels, achieving high sensitivity and purity for CTC recovery while setting performance benchmarks for liquid biopsy and broader clinical diagnostic adoption (24). Advanced enrichment platforms enhance liquid-biopsy diagnostic power. The CTC-iChip merges size filtration with magnetic immunocapture for label-free CTC recovery (25). Di-electrophoresis separates CTCs by dielectric properties, capturing epithelial and mesenchymal phenotypes without markers (26). Instead, photoacoustic flow cytometry detects and isolates rare CTCs in real time via optical-absorption signatures (27). For sEV isolation, density-based ultracentrifugation, size-exclusion filtration and antibody-based immunocapture remain standard approaches, while acoustic nanofilters have recently emerged as efficient high-throughput methods that preserve veicles integrityduring enrichment (28). Microfluidic chips functionalized with anti-CD63/CD81 nanostructures enhance capture specificity (29), the ExoChip platform integrates isolation and analysis in a single step (30), and tangential-flow filtration enables continuous, high-purity sEVs harvesting (31).

**FIGURE 2:**
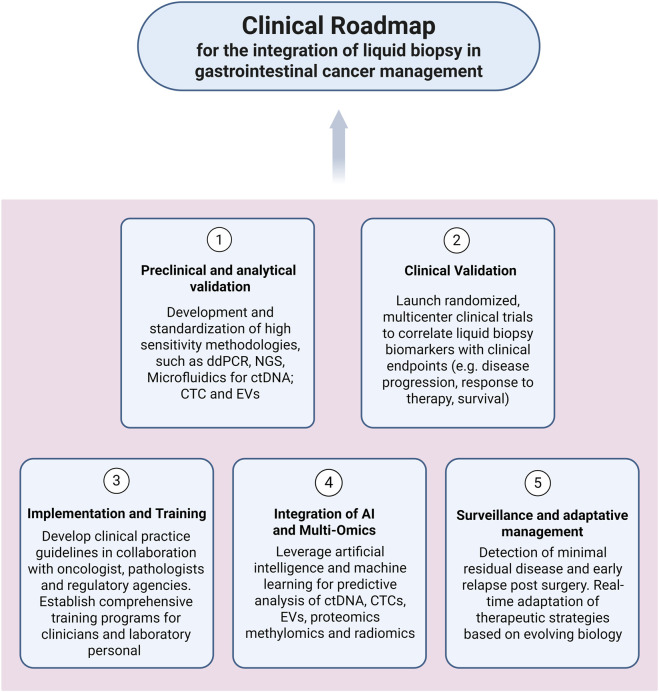
Schematic diagram outlining a unified workflow for isolating circulating tumour cells (CTCs), small extracellular vesicles (sEVs), and circulating tumour DNA (ctDNA) from blood samples. Magnetic beads functionalized with silica or sequence-specific ligands enhance biomarker recovery and shorten processing time. Advances in droplet microfluidics, nanopore sequencing, and integrated microfluidic devices enable sensitive and reproducible detection of rare variants and methylation signatures across all biomarker classes.

As shown in Figure 2, a unified approach to liquid biopsy begins with biological fluids collection and sample preparation, followed by parallel or sequential processing for CTCs, sEVs, and ctDNA. Each biomarker class demands specific pre-analytical and analytical workflows, from magnetic bead capture to microfluidic enrichment and nucleic acid sequencing. Integrated platforms that consolidate isolation, detection, and quantification steps are critical for improving standardization, reducing operator variability, and enabling routine clinical use.

A series of technological innovations has revolutionised the analysis and isolation of circulating biomarkers, improving sensitivity, specificity and operational efficiency. Magnetic beads functionalised with silica or sequence-specific ligands simplify workflows, boost recovery, and cut processing time (32). Nanopore sequencing provides real-time, single-molecule interrogation of ctDNA, detecting rare variants with exceptional sensitivity (33). Instead, droplet microfluidic platforms encapsulate individual fragments, amplify, and sequence them, enabling base-level mutation and methylation profiling (34). Microfluidics and acoustic nanofilters have further enhanced capture specificity; label-free systems now recover both epithelial and mesenchymal CTC phenotypes, overcoming immunoaffinity blind spots (35). While other approaches integrate tangential flow filtration and updated magnetic-bead devices consolidate steps, curtail labour, and lower costs, advantages for resource-limited laboratories (36). Moreover, high-throughput droplet assays and nanopore readers also facilitate continuous tracking of treatment response and MRD, directly informing clinical decisions (37). Automated, microfluidic isolators cement reproducibility and standardisation for routine adoption (38).

## 3 Clinical applications by tumour type

### 3.1 Esophageal cancer

Liquid biopsy is a promising non-invasive tool for managing oesophageal carcinoma (EC), reducing reliance on repeated tissue biopsies in monitoring and treatment guidance (39). In gastroesophageal junction (GEJ) adenocarcinoma treated with pembrolizumab and neoadjuvant chemo-radiotherapy, serial ctDNA analysis effectively tracks treatment response and disease progression; post-therapy ctDNA clearance correlates with higher pathological complete response and better outcomes, while persistence indicates recurrence risk (40). Beyond quantity, ctDNA profiling, including TP53 mutations and methylation, may aid early diagnosis. Singh et al. studied the CAPOX-BETR regimen in advanced HER2-positive GE adenocarcinoma (phase II randomized trial), showing ctDNA-detected amplifications in EGFR, FGFR1, MET, and KRAS correlated with clinical outcomes, supporting its use in personalized treatment (41). Recent data also support the role of ctDNA in minimal residual disease detection and early relapse prediction (42). Ongoing trials like the EXPLORING phase II randomized trial are evaluating ctDNA-guided therapy intensification in ctDNA-positive gastric and GEJ cancers using XELOX, anlotinib, and penpulimab (43). Cell free DNA (cfDNA) levels are elevated in EC versus healthy individuals and carry tumour-specific changes, supporting their role in surveillance (44). In a Randomized controlled trial, the CTC counts, reduced after pre-operative chemotherapy in oesophageal squamous cell carcinoma, associate with improved prognosis, highlighting their utility in treatment assessment (45). Additionally, salivary sEVs rich in tRNA-GlyGCC-5 can distinguish malignant from benign conditions, and combined with real-time sequencing, may enhance early diagnosis and monitoring (46).

### 3.2 Gastric cancer

Gastric cancer (GC) remains a major challenge due to late diagnosis and poor prognosis. Liquid biopsy has transformed non-invasive diagnostics and treatment monitoring, as demonstrated in subsequent studies. In a prospective clinical study, Bai et al. showedthat peritoneal lavage CTCs and ctDNA can predict metachronous peritoneal metastases aftersurgery in patients with advanced GI cancer (47), while Jung et al. confirmed the utility of liquid biopsy for guiding therapy in HER2-positive metastatic GC (48). Izumi et al. validated its use for early-stage GC diagnosis in a prospective study (49), and Modlin et al. demonstrated that multigenomic liquid biopsy markers outperform traditional markers like CgA in neuroendocrine tumours, suggesting relevance in GC (50). Slagter et al. linked higher perioperative ctDNA levels with worse outcomes (CRITICS phase III randomized trial) (51), and phase II trials using CAPOX-bevacizumab-trastuzumab confirmed ctDNA utility in precision oncology (41). In two independent randomized phase III international trials, Lukovic and Rosati provided evidence supporting the feasibility of liquid biopsy markers (52, 53). In GEJ tumours, undetectable ctDNA pre/post-surgery predicted superior survival and correlated with T-cell expansion, highlighting its immunologic role (54). In a phase I study, ctDNA confirmed FGFR2/3 alterations and mirrored response to FGFR inhibitor KIN-3248 (55).

Besides, Cai et al. identified von Willebrand factor-bearing sEVs as diagnostic and therapeutic targets (56), and PD-L1-containing sEVs were linked to poor outcomes post-resection, serving as independent prognostic indicators (57). Contemponary, another study showed that exosomal miR-29b suppressed peritoneal metastases, supporting sEV-based therapy (58), while exosomal miR-92a-3p was also noted as a non-invasive early diagnostic biomarker (59), and BM-MSC-derived exosomes overexpressing miRNA-1228 promoted GC progression via SCAI inhibition (60). Offering new therapeutic avenues, macrophage-derived sEVs from TAMs were shown to promote angiogenesis, metastasis, and resistance (61).

CTCs analysis improved diagnostic accuracy and supported real-time treatment decisions in advanced GC (62). Zhang et al. used CTCs to track trastuzumab resistance in HER2-positive GC (63); Overall, liquid biopsy supports early detection, treatment adjustment, and non-invasive monitoring in GC, with Jung SH et al. reinforcing its value in HER2-targeted therapy (48). ctDNA, CTC, and sEVs aid therapy guidance in neoadjuvant, unresectable, or metastatic GC (52, 53).

### 3.3 Cholangiocarcinoma

Cholangiocarcinoma (CCA), a rare and aggressive bile duct cancer, has benefited from liquid biopsy advances, which offer minimally invasive detection of tumour-specific alterations through ctDNA analysis, especially important given the difficulty of obtaining tissue biopsies (55). ctDNA enables identification of actionable mutations like FGFR2 fusions and IDH1/2 mutations to guide targeted therapy. Garmezy et al. demonstrated ctDNA utility in a phase I clinical trial of the FGFR inhibitor KIN-3248, confirming FGFR2/3 alterations in 63.3% of cases and correlating ctDNA clearance with radiographic response, supporting its role in patient selection and real-time treatment monitoring (55). CTCs, explored by Reduzzi et al., revealed in an observational study, non-conventional CTCs (ncCTCs) lacking epithelial markers, expanding detection capabilities and improving insights into tumour heterogeneity and progression (64). sEVs further advance diagnostic and monitoring strategies, with serum- and utine-derived miR-21 and miR-221 profiles mirroring tumour RNA signatures, while FGFR2 mRNA carried by sEVs supports early detection (65). Gu et al. identified by a prospective observational study a specific exosomal PIWI-interacting RNA (piRNA) signatures, including piR-10506469, piR-20548188, and piR-01856912, as novel diagnostic biomarkers for early detection and personalized care in CCA (66). Together, ctDNA, CTCs, and sEVs reinforce liquid biopsy as a key tool in early diagnosis, monitoring, and precision oncology for CCA.

### 3.4 Colorectal cancer

Colorectal cancer (CRC) ranks third in incidence and second in mortality in high-HDI countries, per 2022 GLOBOCAN (67). Metastases are synchronous in 15%–30% and later develop in 20%–50% of localized cases (68). Carcinogenesis involves APC or TP53 loss, RAS/BRAF/PIK3CA activation, or microsatellite instability (69). EGFR, VEGFR, and HER2 signaling drive progression; HER2 is amplified in 5% of metastases, often with RAS mutations (17%) (70–72). ESMO recommends biomarker profiling before anti-EGFR or anti-VEGFR therapy (68), but pathway mutations often cause resistance (73).

ctDNA enables real-time mutation detection, resistance monitoring, and disease tracking, addressing tissue biopsy limitations, as revealed in the SCRUM-Japan GI-SCREEN and GOZILA studies (74–79). Post-operative ctDNA predicts residual disease and relapse, as shown in prospective and randomized trials (e.g., NEJM 2022, stage II colon cancer study) (80–85); positive status supports adjuvant therapy, while negativity may justify omission (86–88). High baseline MAF or on-treatment VAF predicts poor survival (89–92), and serial ctDNA tracks mutational burden and immune changes in microsatellite-stable CRC (93–96). RAS-wild-type ctDNA indicates anti-EGFR benefit, RAS mutations signal resistance in a non-interventional, uncontrolled multicenter study (97–104). Several randomized phase II trials, including CRONOS, IL VELO, Beyond and CAVE have evaluaed the clearance of RAS, BRAF, or EGFR mutations and supported monoclonal antibody rechallenge (105–117). These findings have subsequently refined adjuvant treatment decisions, as confirmed in both phase II and phase III trials (118–123). In the EVICT (Erlotinib and Vemurafenib in combination trial) and NEW BEACON studies, ctDNA has been used to guide therapy for BRAF V600E and KRAS G12C mutations (124–130). Similary, HER2 (ERBB2) levels in ctDNA inform anti-HER2 tretament decisions and monitor therapeutic response, as demonstrated with pertuzumab in a phase2 trial (131), cetuximab or panitumumab in the NSABP FC-7 a phase Ib study (132), and trastuzumab deruxtecan in the DESTINY-CR01 study (133). Methylation of GRIA4, RARB, VIM, WNT5A, SDC2, SLC8A1, and NPY in ctDNA correlates with poor prognosis and may aid early detection (134–136). In the same way, cfDNA-based screening models like GALNT9/UPF3A show high sensitivity and specificity (137). Contemporary, in a multicenter clinical study, Whang et al. reported that the MethyDT test (NTMT1/MAP3K14-AS1) outperforms SEPT9 for CRC diagnosis (138), offering better compliance, though further validation is needed (139). Blood-based MSI burden from ctDNA predicts immunotherapy response (140), though distinguishing tumour from immune DNA remains challenging (141).

CTCs correlate with metastasis, invasiveness, and prognosis in CRC (142, 143), identifying patients for intensified treatment based on FOLFOXIRI and bevacizumab versus FOLFOX, in a randomised phase III VISNÚ-1 trial (144), and in an observational cohort study (145). CTC enumeration assesses surgery or stent outcomes (146, 147), while Wu et al. in an experimental study demonstratedthat the detection in peritoneal lavage predicts poor outcomes (148). Mesenchymal CTCs signal relapses and high mortality (149, 150). CTCs also correlate with immune dysfunction in MRD and worse survival (151–153), possibly due to MMP-2-mediated immunosuppression (154), though some studies report limited added value (155). EVs offer alternative biomarkers; tumour-derived EVs promote progression, and endothelial EVs predict survival in metastatic CRC (156). CRC-plasma EVs reprogram monocytes and differ by disease stage (157). sEVs associated miRNAs, such as miR-19b, miR-21, miR-222, and miR-92a contribute to early diagnosis with high miR-222 levels predicting worse survival (158), while low sEV-miR-193a-5p is associated with nodal spread (159). Exosomal circ-133 rises with disease stage (160), and circ-HMGCS1 drives invasion via the circ-HMGCS1/miR-34a-5p/SGPP1 axis (161). A multicenter study identified a five-miRNA fecal signature (miR-1246, miR-607-5p, miR-6777-5p, miR-4488, miR-149-3p), with potential to improve non-invasive CRC screening (162).

### 3.5 Pancreatic cancer

Pancreatic cancer (PC) remains a top cause of cancer mortality, with about 467,000 deaths in 2022 and a 10% survival rate (67, 163). Late diagnosis limits curative options, highlighting the need for early biomarkers. KRAS-mutant ctDNA is scarce and error-prone; combining it with serum protein markers improves diagnostic accuracy (164). Tumour-derived ctDNA is shorter than benign cfDNA, particularly in early-stage PC (165). In PDAC, ctDNA detects BRCA2 mutations for PARP inhibitor use and clonal KRAS/GNAS alterations (166, 167). KRAS-mutant ctDNA signals poor prognosis, while wild-type status relates to better survival, though it does not predict immunotherapy response. KRAS G12D/V mutations expand T-reg cells and suppress antitumour immunity, especially G12V (168). Elevated neutrophil-to-lymphocyte ratios correlate with ctDNA presence, linking inflammation and tumour burden. Serial ctDNA declines during effective therapies correlate with improved survival in advanced disease (169–171). Particularly, Pant et al underligthed the use of ELI-002P vaccine to recude the ctDNA in several patients affected by PC enrolled in the phase 1 AMPLIFY-201 trial (172). Postoperative ctDNA drop predicts longer survival; cfDNA fragmentomics supports this trend (173, 174). Despite limited yield, ctDNA retains prognostic value post-chemotherapy; pre-op ctDNA still reflects tumour status in a non-randomized controlled trial (175). Methylation assays (HOXD8, POU4F1) of circulation tumor DNA enhance prognostication in metastatic PC by a *post hoc* analyses of two clinical trials (176). In a prospective observational study, named “PASEA” was detected KRAS mutations in 62.4% of PDAC, with ctDNA clearance marking stability and reappearance signaling progression (177). CTCs predict drug response and survival in advanced PDAC (178), track treatment response and early metastasis, and are detectable in early stages via microfluidic devices (179–181). EVs also hold diagnostic promise, though differentiation from benign EVs is needed. A three-module PPI model identified LEP and SSTR5 as key regulators with prognostic value (182). A digital ELISA test (DEST) showed elevated MUC5AC in EVs predicts IPMN progression to carcinoma (183). EVs-TFs trigger pro-thrombotic states, driving progression and chemo-resistance, and independently predict mortality (184). GPC1 and CD82 markers in EVs may support diagnosis (185). EVs RNA studies show miR-200 family upregulation promotes EMT and metastasis, with high diagnostic accuracy (186). PDAC EVs also deliver miR-155-5p, which activates NF-κB, suppresses EHF, and drives invasiveness (187). In a multicenter case-control study, six dysregulated exosomal miRNAs (including miR-21-5p, miR-223-3p) show diagnostic value, especially with CA19-9, though post-treatment dynamics remain unclear (188). A three-miRNA signature (PPP1R12A, SCN7A, SGCD) predicted poor survival (189). Combining cf-miRNA and exo-miRNA yielded a 13-miRNA signature for early detection, even in low CA19-9 cases (multicenter cohort study) (190). Two diagnostic plasma panels include five miRNAs (miR-215-5p, miR-122-5p, miR-192-5p, miR-30b-5p, miR-320b) (191) and three (hsa-miR-1246, hsa-miR-205-5p, hsa-miR-191-5p) (192). CA19-9 remains a standard marker, but protein panels (193), inflammatory markers (FAR, FPR, FLR) (194), or ctDNA (174) enhance its diagnostic range and detect non-threshold cases. The glycan sTRA, combined with CA19-9, may predict chemo-resistance (195). Autotaxin, secreted by CAFs, mediates treatment resistance and tumour growth post-TGFβ inhibition, suggesting value in monitoring therapy (196). The NETest, a multigene blood test, aids early detection and monitoring of neuroendocrine cancers (50, 197, 198). Despite progress, identifying reliable biomarkers for early PC detection and treatment response remains challenging.

### 3.6 Liver cancer

Liver cancer is the third leading cause of cancer-related death globally, with 865,000 new cases in 2022 (67). Hepatocellular carcinoma (HCC), mostly caused by chronic HBV or HCV infection, represents 75%–85% of cases (199). Due to poor early detection, diagnosis often occurs at advanced stages (200). Liquid biopsy is gaining value for early diagnosis and monitoring. The Hepa-AiQ ctDNA methylation test outperformed AFP and DCP for early-stage HCC and relapse prediction, though limited to CHB/LC-related cases in Chinese patients (prospective validation study) (201). In the PETAL phase Ib study, D.J. Pinato and colleagues demonstrated that ctDNAeffectively tracked responses to neoantigen vaccines and revealed tumor heterogeneity; however, the immune response was insufficient to fully eradicate residual disease (202). In another phase II clinical trial, Y.Xia et al. estabilished that the changes in ctDNA levels reflected radiological response to TACE with PD-1 inhibitors (203) and post-op increases predicted recurrence during immunosuppressive therapy (204). In HBV-related cases, vh-DNA tracked tumour burden and recurrence risk but lacks general applicability (205). cfDNA concentrations post-resection independently predicted recurrence better than AFP (206). cSMART-detected mutations (TERT, TP53, CTNNB1), combined with AFP, AFP-L3, and PIVKA-II, created a model superior to AFP alone, especially for early HCC (207). The mt-HBT test, combining cfDNA methylation markers, AFP, and gender, showed 88% overall and 82% early-stage sensitivity, outperforming AFP and GALAD (208). The PreCar Score, based on cfDNA features, enhanced detection in non-cirrhotic, HBV-related cases, especially when paired with ultrasound (209). CTC counts and mesenchymal traits predicted recurrence risk and informed resection strategy (210), while the anterior approach reduced intraoperative CTC spread and early relapse (211). CTC-based models, incorporating size, nodule count, and MVI, accurately predicted recurrence (212), and high CTC levels before/after surgery indicated poorer survival and metastasis risk (213). EV size >145.65 nm before TACE was associated with worse prognosis (214). sEV proteins like A2MG and PIGR showed better diagnostic accuracy than AFP, while others (Fetuin-A, Meprin A) indicated progression in the SORAMIC trial study (215). In non-viral HCC, EV markers (GPX3, ACTR3, ARHGAP1) predicted SIRT and sorafenib outcomes (216). EV lncRNAs such as SENP3-EIF4A1, FAM72D-3, EPC1-4, and a panel (MALAT1, DLEU2, HOTTIP, SNHG1) showed diagnostic and prognostic potential (217, 218). A combined EV purification and RT-ddPCR test achieved high sensitivity and specificity in early HCC detection (219). MYCN correlated with liver function and fibrosis, outperforming AFP in predicting progression (220). A five-protein panel (OPN, GDF15, NSE, TRAP5, OPG) effectively detected early-stage HCC (221), and a seven-autoantibody panel showed greater sensitivity than AFP (222). Spectroscopy proved useful for early detection in obese cirrhotic patients where ultrasound fails (223), and platelet mRNA markers have been proposed for early-stage HCC detection (224).

## 4 Clinical decision framework: matching liquid biopsy tools to clinical objectives

To translate emerging evidence into actionable clinical strategies, we propose a decision-oriented framework that aligns each liquid biopsy modality ctDNA, CTCs, and EVs with specific oncologic goals in gastrointestinal (GI) cancers. For instance, EV-based profiling, especially in saliva or plasma, offers promise for early detection or screening, particularly when combined with miRNA signatures. Furthermore, sEVs represent a unique biomarker class in liquid biopsy, offering molecular cargo that captures complex tumor biology beyond genetic mutations alone. Unlike ctDNA, which primarily reflects tumor-specific genetic alterations, and CTCs, which provide phenotypic and genomic information on intact circulating tumor cells, EVs carry a diverse set of bioactive molecules including miRNAs, proteins, lipids, and metabolites. This cargo influences tumor progression, immune evasion, and metastatic niche formation, thus providing insights into tumor microenvironment interactions and systemic disease processes. For example, specific EV-derived miRNAs such as miR-21, miR-29b, and miR-92a-3p have been linked to tumor growth, chemoresistance, and prognosis in gastrointestinal cancers, while proteins like PD-L1 carried on EVs correlate with immune checkpoint modulation and therapy response. These molecular signatures offer distinct clinical advantages, especially in scenarios where ctDNA levels are low or CTC capture is challenging, such as in early-stage disease or certain upper GI cancers. Furthermore, sEVs are highly stable in bodily fluids, making them suitable for repeated sampling and longitudinal monitoring. Emerging evidence also supports their role in predicting treatment outcomes and immune responses, thereby complementing the information obtained from ctDNA and CTC analyses and enriching personalized oncology strategies. Conversely, ctDNA analysis via serial plasma sampling is the most robust tool for MRD detection, treatment response monitoring, and molecular relapse prediction. CTC enumeration and phenotyping, on the other hand, may be particularly informative for predicting metastatic spread, immune evasion, and drug resistance, especially in cancers such as colorectal and pancreatic carcinoma.

The choice of biomarker is also informed by tumour location and disease extent. In upper GI malignancies (esophageal, gastric, cholangiocarcinoma), CTCs and sEVs often yield higher diagnostic utility due to anatomical sampling limitations. In lower GI cancers (colorectal, pancreatic, hepatocellular carcinoma), ctDNA tends to be more abundant and clinically actionable, especially in the metastatic setting. Finally, advanced-stage disease or patients under active systemic therapy may benefit most from real-time ctDNA tracking, while drug resistance can be further evaluated by combining ctDNA mutation profiling with dynamic CTC analysis.

### 4.1 Overview of clinical trials

A systematic analysis of ongoing clinical trials was conducted using the ClinicalTrials.gov database (accessed on 22 September 2025). Each gastrointestinal malignancy (oesophageal, gastric, colorectal, pancreatic, hepatic, and biliary tumours) was searched in combination with terms related to liquid biopsy (CTCs, ctDNA, EVs, and “liquid biopsy”). The total number of active studies is presented in Table 1, while the complete list, including identifiers, project titles, and URLs, is provided in Supplementary Table S1.

**TABLE 1:** Number of ongoing clinical trials on liquid biopsy in gastrointestinal cancers (ClinicalTrials.gov, accessed 22 September 2025).

Type of tumor	ctDNA	CTCs	EVs	Liquid biopsy
Oesophageal Cancer	10	1	0	8
Gastric Cancer	15	3	0	6
Cholangiocarcinoma	4	0	0	0
Colorectal Cancer	37	7	2	15
Pancreatic Cancer	12	2	0	8
Liver Cancer	6	1	0	5

The distribution of ongoing clinical trials indicates significant trends in the translational adoption of liquid biopsy in gastrointestinal cancers. Colorectal cancer emerges as the leading field, with a predominance of ctDNA-based studies, reflecting its central role in the identification of minimal residual disease, therapeutic monitoring, and clinical decision-making. Gastric and oesophageal cancers also show a growing number of ctDNA- and liquid biopsy-oriented studies, in line with their clinical necessity to improve early diagnosis and response assessment. In contrast, cholangiocarcinoma and liver cancer remain underrepresented, with only a handful of ctDNA-focused initiatives, underscoring the limited clinical translation of liquid biopsy in these settings. In detail, studies specifically investigating EVs are virtually non-existent, suggesting that while preclinical evidence is expanding, its incorporation into large-scale clinical protocols is still in its early stages. On an overall basis, the current study landscape highlights the greater clinical readiness of ctDNA compared to CTCs and VEs, who remain in the early stages of translational validation.

## 5 From bench to bedside: a clinical integration roadmap for liquid biopsy in GI oncology

**FIGURE 3:**
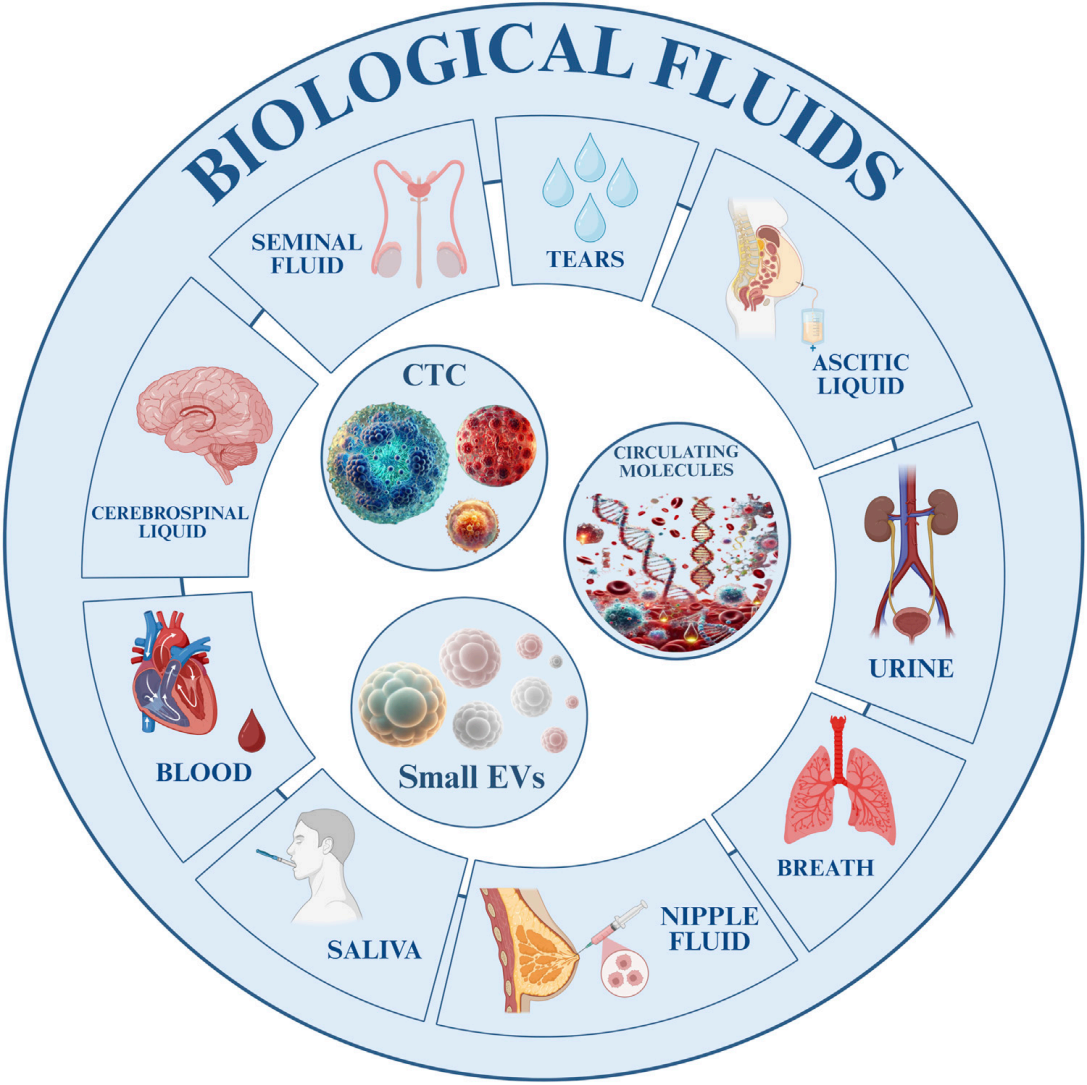
Flowchart illustrating the proposed clinical roadmap for integrating liquid biopsy in gastrointestinal cancer management. The diagram outlines five sequential phases: (1) analytical validation; (2) clinical validation and threshold definition; (3) guideline development and training; (4) multi-omics and AI integration; (5) adaptive surveillance and therapeutic adjustment.

Liquid biopsy holds strong promise across GI cancers, but its clinical translation remains incomplete. To move from experimental utility to standard of care, a structured roadmap is needed one that aligns assay development, regulatory validation, and clinical adoption. We propose a five-phase integration model to guide the systematic implementation of liquid biopsy platforms across GI malignancies (Figure 3). This framework emphasizes harmonization of technologies, validation through clinical endpoints, and interdisciplinary collaboration among oncologists, pathologists, and laboratorians.

### 5.1 Phase 1: analytical and preclinical validation

The first step toward clinical translation of liquid biopsy is the development of high-fidelity, reproducible assays for ctDNA, CTCs, and EVs. A critical requirement at this stage is the implementation of standardized workflows, capable of reducing inter-laboratory variability and ensuring clinical comparability.

Recent studies in various cancer types have shown the feasibility of standardized liquid biopsy workflows. For example, Sathyanarayana et al. reported an automated cfDNA extraction and quantification protocol validated across multiple centers, ensuring reproducibility and minimizing pre-analytical variability (225). The International Society of Liquid Biopsy (ISLB) recently issued minimal quality control requirements for ctDNA analysis, stressing harmonization across pre-analytical, analytical, and post-analytical phases (226). Pantel and Alix-Panabières highlighted key barriers to CTC adoption and advocated inter-laboratory trials with robust benchmarking to speed translation (227). In the pre-analytical setting, Grölz et al. showed that collection tubes, transport time, and storage conditions critically affect cfDNA integrity and downstream analyses (228).

Building on these experiences, we propose a GI-specific analytical workflow, aimed at addressing the unique challenges of GI tumors:

• Pre-analytical standardization. Use of cfDNA-stabilizing blood collection tubes to reduce leukocyte lysis. Strict limits for transport (<24 h) and processing times, under controlled temperature. Defined centrifugation protocols (two-step processing with standardized speeds).
• Controlled extraction and enrichment. Automated bead-based methods for cfDNA isolation with internal QC metrics (yield, size distribution). Microfluidic or immunoaffinity platforms for reproducible CTC and EV enrichment. Validation of devices such as ExoChip or CTC-iChip in GI-specific clinical settings.
• Analytical performance benchmarking
• Definition of thresholds for sensitivity, specificity, and limit of detection (LOD) through the use of reference standards and spike-in controls. Inclusion of both positive and negative process controls in every analytical run.
• Inter-laboratory harmonization. Establishment of ring trials among reference centers to assess reproducibility of ctDNA allele frequency quantification and CTC counts. Development of shared databases and consensus reporting templates.
• Bioinformatics and reporting. Adoption of error-corrected sequencing pipelines, including molecular barcoding, to reduce false positives. Transparent reporting of quality metrics (e.g., read depth, fragment size distribution, variant allele frequency confidence). Harmonization of output into clinically interpretable reports for integration into tumor boards.

### 5.2 Phase 2: clinical validation

This phase focuses on demonstrating the correlation between liquid biopsy metrics and meaningful clinical endpoints:

• Launch of prospective, multi-center trials to assess ctDNA, CTCs, and EVs in early diagnosis, treatment response, and MRD detection.
• Definition of actionable thresholds (e.g., ctDNA mutant allele frequency, CTC count cutoffs).
• Cross-comparison with conventional markers such as CEA, CA19-9, AFP, and radiologic imaging.
• Integration with histology, tumour stage, and therapy type to refine biomarker interpretation.

### 5.3 Phase 3: implementation, and training

For clinical integration, three parallel initiatives must occur:

• Development of practical guidelines, consensus statements, and diagnostic algorithms for biomarker use in specific GI tumour types.
• Training programs for clinicians, lab personnel, and oncology teams on interpretation and use of liquid biopsy data.
• Inter-laboratory standardization networks to ensure reproducibility, quality assurance, and data interoperability.

### 5.4 Phase 4: integration of AI and multi-omics

As datasets grow in complexity, artificial intelligence and machine learning will be essential to:

• Integrate liquid biopsy data with proteomics, methylomics, radiomics, and clinical variables.
• Develop predictive models for recurrence, response, and resistance.
• Identify novel biomarker signatures using pattern recognition from high-dimensional data.

### 5.5 Phase 5: surveillance and adaptive management

The final phase positions liquid biopsy as a cornerstone of precision surveillance:

• Use of serial ctDNA and CTC analysis to detect early relapse and MRD.
• Real-time biomarker feedback to guide therapy escalation, de-escalation, or rechallenge strategies.
• Incorporation into adaptive trial designs and tumour board decision-making.

## 6 Limitations and future directions

Recent advances in liquid biopsy research are promising; however, significant limitations continue to constrain the robustness and generalizability of the evidence in GI oncology. Many studies involve small and heterogeneous patient cohorts, which limits statistical power and hampers meaningful subgroup analyses, particularly for less common malignancies such as cholangiocarcinoma, where data remain notably sparse. Methodological variability remains a critical barrier. Differences in pre-analytical procedures—including blood collection tubes, centrifugation protocols, and storage conditions—combined with inconsistencies in analytical platforms, such as sequencing technologies, PCR assays, and enrichment methods, contribute to significant inter-laboratory variability. This lack of standardization hinders the establishment of clinically relevant thresholds for biomarkers like ctDNA allele frequency, CTC counts, and EV signatures.

Furthermore, the geographic and institutional concentration of existing studies limits external validity, as much of the evidence originates from single-centre investigations or cohorts from East Asia and selected European institutions. These limitations raise concerns about the broader applicability of findings across diverse populations and healthcare systems. Additionally, much of the current data is descriptive or exploratory. Although retrospective analyses and early-phase prospective trials offer valuable proof-of-concept insights, large, randomized, multi-centre studies demonstrating improvements in overall survival, progression-free survival, or cost-effectiveness are still scarce.

Biological complexities also pose substantial challenges to clinical translation. Intratumourally heterogeneity, clonal evolution, and variability in biomarker shedding contribute to false negatives and inconsistent results, while distinguishing tumour-derived signals from background circulating material remains particularly difficult in early-stage disease when biomarker abundance is low. Looking forward, the field must prioritize harmonized protocols, broad international collaboration, and the incorporation of artificial intelligence–driven analytic frameworks. Only through rigorously designed, globally representative clinical trials can liquid biopsy transition from an experimental adjunct to a validated, standardized component of routine oncological care. Addressing economic factors, regulatory heterogeneity, inter-laboratory variability, and educational needs in parallel with technological and clinical advances is essential to ensure the effective integration of liquid biopsy into everyday clinical practice.

## 7 Conclusion

Liquid biopsy offers a sensitive, minimally invasive complement to tissue sampling in GI malignancies. Its components CTCs, ctDNA, and EVs capture intratumour heterogeneity, support early detection and enable real-time therapeutic monitoring (229). In EC and GC, combining ctDNA with CTCs enumeration refines neoadjuvant decision-making (4), while early detection of resistance allows rapid therapy adjustment. Protein- or *miRNA*-enriched sEVs sharpen prognosis and may predict immunotherapy benefit (230–232). CCA presents unique diagnostic and therapeutic challenges, often due to the difficulty of obtaining adequate tissue samples. In this setting, ctDNA profiling for actionable alterations in genes such as FGFR and IDH not only circumvents the limitations of tissue biopsy but also informs the selection of targeted therapies. Longitudinal monitoring of ctDNA can guide modifications in dosage or therapeutic agents over the course of treatment, supporting a more adaptive and responsive approach to disease management (233, 234). CRC has been at the forefront of liquid biopsy adoption, with post-operative ctDNA detection serving as a highly sensitive indicator of MRD. Dynamic changes in ctDNA mutation profiles can herald impending relapse, while the emergence of Neo-RAS wild-type status may reopen eligibility for anti-EGFR therapies, thereby expanding treatment options (235, 236). Furthermore, analysis of sEVs cargo has been shown to provide additional risk stratification for metastatic disease, particularly in cases where conventional markers are inconclusive (237). In PC, the diagnostic sensitivity of liquid biopsy is enhanced by integrating ctDNA analysis with serum protein markers or by employing fragment omics approaches to detect subclinical disease. The identification of KRAS-associated regulatory T cell enrichment and chemoresistance-associated circulating tumour-initiating cells provides valuable insights for guiding immunological and pharmacological interventions (238, 239). Exosomal *miRNAs*—*miR-200* family, *miR-155-5p*—augment diagnostic and prognostic panels (240). HCC studies show ctDNA methylation assays (e.g., HepaAiQ) and virus-host DNA hybrids enhance early detection, while combining ctDNA with AFP, AFP-L3 and PIVKA-II yields superior accuracy (201, 208). Counting, CTCs alongside MET markers and EVs-derived molecules yields strong prognostic value and sharper post-operative surveillance. Yet broad clinical use of liquid biopsy still depends on assay standardisation, inter-laboratory reproducibility and the management of biomarker heterogeneity. In addition to the discussion above, Table 2 provides a comprehensive summary of the key studies referenced throughout the manuscript, detailing the application of liquid biopsy techniques, across a spectrum of GI cancers such as oesophageal, gastric, cholangiocarcinoma, colorectal, pancreatic, and liver malignancies. Each entry in the table specifies the tumour type, the liquid biopsy component investigated, and the corresponding references that support the diagnostic, prognostic, and therapeutic insights discussed. This compilation further underscores the expanding evidence base for the integration of liquid biopsy into routine cancer management and highlights its potential to transform clinical practice as standardization and validation efforts advance. Large, prospective, multi-center trials are needed to validate biomarkers and cement uniform protocols. Growing data nevertheless show that liquid biopsy improves early detection, patient stratification, treatment guidance and disease monitoring in GI cancers. As ongoing research resolves current obstacles, this sensitive, dynamic and non-invasive approach is likely to become a mainstay of GI oncology, raising the standard of care and patient outcomes.

**TABLE 2:** Summary table presenting the key studies cited throughout the manuscript, highlighting liquid biopsy techniques—including ctDNA, CTCs, EVs and generally liquid biopsy, across various GI cancers. Each entry specifies tumour type, liquid biopsy component studied, and the associated references supporting the discussed diagnostic, prognostic, therapeutic insights, and promising biomarkers. This compilation underscores the growing evidence base for integrating liquid biopsy into cancer management.

Type of tumour	ctDNA	CTCs	EVs	Liquid biopsy
Esophageal Cancer (EC)	Post-therapy ctDNA clearance predicts better response and survival in GEJ adenocarcinoma (40) ctDNA profiling (TP53, EGFR, KRAS) aids personalized treatment (41)	Reduced CTC counts post-chemotherapy correlate with improved prognosis (45)	Salivary sEVs with tRNA-GlyGCC-5 signature distinguish malignant from benign lesions, potential early diagnostic tool (46)	
Gastric Cancer (GC)	Peritoneal lavage ctDNA and CTCs predict metastases post-surgery (47) Undetectable ctDNA pre/post-surgery predicts superior survival (54)	CTCs track trastuzumab resistance in HER2+ metastatic GC (63)	Von Willebrand factor-bearing EVs as diagnostic markers (56) PD-L1+ sEVs linked to poor prognosis (57) Exosomal miRNAs (miR-29b, miR-92a-3p, miR-1228) regulate metastasis and progression (58–61)	Monitoring HER2-positive metastatic gastric cancer therapy (48, 49)
Cholangiocarcinoma (CCA)	ctDNA detects actionable FGFR2 fusions and IDH1/2 mutations, guides targeted therapy (55)	Non-conventional CTCs lacking epithelial markers reveal tumour heterogeneity (64)	EVs miR-21, miR-221, and piRNA signatures serve as early diagnostic biomarkers (65, 66)	
Colorectal Cancer (CRC)	ctDNA predicts post-op relapse and guides adjuvant therapy (74–79) Prospective trials validate ctDNA for minimal residual disease (80–85)	CTCs associate with metastasis and prognosis (142, 143) CTC-based stratification for intensive chemo regimens (144)	sEVs miRNAs (miR-19b, miR-21, miR-222, miR-92a) support early diagnosis (156–159) circRNAs linked to invasion and disease progression (160)	Five-miRNA fecal signature (miR-1246, miR-607-5p, miR-6777-5p, miR-4488, miR-149-3p), showing potential for improving non-invasive CRC screening (162)
Pancreatic Cancer (PC)	KRAS-mutant ctDNA combined with protein markers improves diagnosis (164–171) ctDNA methylation (HOXD8, POU4F1) predicts prognosis (176)	CTCs predict drug response and early metastasis (178–181)	EVs markers (MUC5AC, GPC1, CD82) and exosomal miRNAs (miR-200 family, miR-155-5p) as diagnostic and prognostic tools (182–192)	Plasma panels with specific miRNAs improve diagnosis (191, 192). CA19-9 gains accuracy combined with protein/inflammatory markers or ctDNA (174, 193, 194). sTRA predicts chemoresistance with CA19-9 (195). Autotaxin linked to therapy resistance (196). NETest aids early detection and monitoring of neuroendocrine cancers (50, 197, 198)
Liver Cancer (Hepatocellular Carcinoma, HCC)	ctDNA methylation assays (e.g., Hepa-AiQ) combined with AFP, AFP-L3, PIVKA-II for early detection (201–205)	CTC counts and phenotypes predict recurrence risk and metastasis (210–213)	EVs proteins and lncRNAs (A2MG, PIGR, MALAT1, SENP3-EIF4A1) enhance diagnosis and prognosis (214–219)	cfDNA post-resection predicts recurrence better than AFP (206). Multi-marker models (cSMART (207), mt-HBT (208), PreCar (209)) improve early HCC detection MYCN (220), protein/autoantibody panels (221, 222), and spectroscopy (223) outperform AFP in specific contexts

Liquid biopsy has emerged as a transformative tool in GI oncology, enabling minimally invasive diagnosis, real-time monitoring, and dynamic treatment adaptation. While ctDNA, CTCs, and EVs have each demonstrated clinical relevance, their true potential will be unlocked through integration with multi-omics and AI-driven analytics. Such approaches will allow the simultaneous incorporation of genomic, epigenomic, transcriptomic, proteomic, and radiomic features into predictive models, refining patient stratification and guiding precision therapies. Looking forward, certain biomarkers appear particularly promising in specific GI cancers: ctDNA for minimal residual disease detection in colorectal and pancreatic cancers; CTCs for predicting metastasis and therapeutic resistance in esophageal and gastric cancers; EV-derived signatures (miRNAs, proteins) for early detection and immunomodulation in gastric and liver cancers; and ctDNA for actionable mutations in cholangiocarcinoma and hepatocellular carcinoma. As large-scale prospective studies validate these applications and standardization improves, liquid biopsy—augmented by multi-omics and AI—will become a cornerstone of precision oncology, offering more tailored and adaptive management strategies for patients with GI malignancies.

This study highlights several promising biomarkers reported in Table 2 with potential applications in early diagnosis, prognosis, and as therapeutic targets in GIcancers. Given the continuous evolution and dynamic nature of this research field, these biomarkers represent valuable tools not only for improving clinical decision-making but also for guiding the development of innovative therapeutic strategies.
