# Supplementary material for: Liquid biopsy in gastrointestinal oncology: clinical applications and translational integration of ctDNA, CTCs, and sEVs
Source: Oncol Rev. 2025 Oct 20;19:1702932. doi: 10.3389/or.2025.1702932 (PMC12580207; doi:10.3389/or.2025.1702932)
Supplement: Supplementary file 1 [file Supplementaryfile1.docx]

Supplementary Materials

**Supplementary Table 1S.** List of ongoing clinical trials on liquid biopsy in gastrointestinal cancers (ClinicalTrials.gov, accessed 22 September 2025). The table reports for each study the identifier code, project title, and URL. Abbreviations: ctDNA, circulating tumor DNA; CTCs, circulating tumor cells; EVs, extracellular vesicles.

|  | **Codice** | **Nome** | **URL** |
| --- | --- | --- | --- |
| OESOPHAGEAL CANCER - ctDNA | NCT03653052 | Do Changes in ctDNA Predict Response for Patients With Oesophageal Cancer Receiving Durvalumab | <https://clinicaltrials.gov/study/NCT03653052> |
|  | NCT06143748 | Combination of Cadonilimab and Chemoradiotherapy in Esophageal Cancer (EC-CRT-006) | <https://clinicaltrials.gov/study/NCT06143748> |
|  | NCT03421288 | Study of Atezolizumab + FLOT vs. FLOT Alone in Patients With GC/GEJ and High Immune Responsiveness | <https://clinicaltrials.gov/study/NCT03421288> |
|  | NCT03764553 | Liposomal iRInotecan, Carboplatin or oXaliplatin for Esophagogastric Cancer | <https://clinicaltrials.gov/study/NCT03764553> |
|  | NCT04046575 | Radiation Dose Intensification With Accelerated Hypofractionated Intensity Modulated Radiation Therapy and Concurrent Carboplatin and Paclitaxel for Inoperable Esophageal Cancer | <https://clinicaltrials.gov/study/NCT04046575> |
|  | NCT05520619 | Combination of Tislelizumab and Chemoradiotherapy in Esophageal Cancer (EC-CRT-002) | <https://clinicaltrials.gov/study/NCT05520619> |
|  | NCT03712566 | Multi-Omic Assessment of Squamous Cell Cancers Receiving Systemic Therapy | <https://clinicaltrials.gov/study/NCT03712566> |
|  | NCT05650827 | Feasibility and Effect of Resistance Training and Protein Supplementation in Patients With Advanced Gastroesophageal Cancer | <https://clinicaltrials.gov/study/NCT05650827> |
|  | NCT03505320 | A Study of Zolbetuximab (IMAB362) in Adults With Gastric Cancer | <https://clinicaltrials.gov/study/NCT03505320> |
|  | NCT04196465 | Phase II Study of Neoadjuvant Immune Checkpoint Inhibitor in Patients With Resectable Gastrointestinal Cancers | <https://clinicaltrials.gov/study/NCT04196465> |
| OESOPHAGEAL CANCER - CTCs | NCT02037048 | FOLFOX-6 Induction Chemotherapy Followed by Esophagectomy and Post-operative Chemoradiotherapy in Patients With Esophageal Adenocarcinoma | <https://clinicaltrials.gov/study/NCT02037048> |
| OESOPHAGEAL CANCER – Liquid Biopsy | NCT03835663 | The Bacterial Composition of the Stomach in Reflux Disease | <https://clinicaltrials.gov/study/NCT03835663> |
|  | NCT04248582 | Cryotherapy for Locally Advanced Esophageal Cancer | <https://clinicaltrials.gov/study/NCT04248582> |
|  | NCT03712566 | Multi-Omic Assessment of Squamous Cell Cancers Receiving Systemic Therapy | <https://clinicaltrials.gov/study/NCT03712566> |
|  | NCT05107219 | GCC Agonist Signal in the Small Intestine | <https://clinicaltrials.gov/study/NCT05107219> |
|  | NCT02381561 | Ropidoxuridine in Treating Patients With Advanced Gastrointestinal Cancer Undergoing Radiation Therapy | <https://clinicaltrials.gov/study/NCT02381561> |
|  | NCT02521285 | Aspirin in Preventing Disease Recurrence in Patients With Barrett Esophagus After Successful Elimination by Radiofrequency Ablation | <https://clinicaltrials.gov/study/NCT02521285> |
|  | NCT05421689 | Autologous Muscle Derived Cells for Treatment of Tongue Dysphagia | <https://clinicaltrials.gov/study/NCT05421689> |
|  | NCT03676478 | Assessing Timing of Enteral Feeding Support in Esophageal Cancer Patients on Muscle functTion and Survival | <https://clinicaltrials.gov/study/NCT03676478> |
| GASTRIC CANCER - ctDNA | NCT05029869 | Monitoring Minimal Residual Disease in Gastric Cancer by Liquid Biopsy Study Description | <https://clinicaltrials.gov/study/NCT05029869> |
|  | NCT06893133 | ctDNA-MRD Monitoring After Resection in Gastric Cancer | <https://clinicaltrials.gov/study/NCT06893133> |
|  | NCT04084249 | ctDNA-guided Surveillance for Stage III CRC, a Randomized Intervention Trial | <https://clinicaltrials.gov/study/NCT04084249> |
|  | NCT06939439 | Using ctDNA to Guide Treatment Decisions for Stage III Gastric Cancer | <https://clinicaltrials.gov/study/NCT06939439> |
|  | NCT06940778 | LIQUID BIOPSY FOCUSING ON CIRCULATING TUMOR CELLS AND CIRCULATING TUMOR DNA AS PRECISION MEDICINE IN GASTROINTESTINAL TUMORS | <https://clinicaltrials.gov/study/NCT06940778> |
|  | NCT03421288 | Study of Atezolizumab + FLOT vs. FLOT Alone in Patients With GC/GEJ and High Immune Responsiveness | <https://clinicaltrials.gov/study/NCT03421288> |
|  | NCT04817826 | TremelImumab aNd Durvalumab For the Non-operatIve Management (NOM) of MSI-high Resectable GC/GEJC. | <https://clinicaltrials.gov/study/NCT04817826> |
|  | NCT06157216 | Minimal Residual Disease-guided Adjuvant Therapy for Gastric Cancer | <https://clinicaltrials.gov/study/NCT06157216> |
|  | NCT04931654 | A Study to Assess the Safety and Efficacy of AZD7789 in Participants With Advanced or Metastatic Solid Cancer | <https://clinicaltrials.gov/study/NCT04931654> |
|  | NCT05836584 | Testing Immunotherapy (Atezolizumab) With or Without Chemotherapy in Locoregional MSI-H/dMMR Gastric and Gastroesophageal Junction (GEJ) Cancer | <https://clinicaltrials.gov/study/NCT05836584> |
|  | NCT05190445 | Cinrebafusp Alfa in Combination With Ramucirumab and Paclitaxel in HER2-High Gastric or GEJ Adenocarcinoma and in Combination With Tucatinib in HER2-Low Gastric or GEJ Andenocarinoma | <https://clinicaltrials.gov/study/NCT05190445> |
|  | NCT05650827 | Feasibility and Effect of Resistance Training and Protein Supplementation in Patients With Advanced Gastroesophageal Cancer | <https://clinicaltrials.gov/study/NCT05650827> |
|  | NCT03505320 | A Study of Zolbetuximab (IMAB362) in Adults With Gastric Cancer | <https://clinicaltrials.gov/study/NCT03505320> |
|  | NCT02264678 | Ascending Doses of Ceralasertib in Combination With Chemotherapy and/or Novel Anti Cancer Agents | <https://clinicaltrials.gov/study/NCT02264678> |
|  | NCT04196465 | Phase II Study of Neoadjuvant Immune Checkpoint Inhibitor in Patients With Resectable Gastrointestinal Cancers | <https://clinicaltrials.gov/study/NCT04196465> |
| GASTRIC CANCER - CTCs | NCT06940778 | LIQUID BIOPSY FOCUSING ON CIRCULATING TUMOR CELLS AND CIRCULATING TUMOR DNA AS PRECISION MEDICINE IN GASTROINTESTINAL TUMORS | <https://clinicaltrials.gov/study/NCT06940778> |
|  | NCT02037048 | FOLFOX-6 Induction Chemotherapy Followed by Esophagectomy and Post-operative Chemoradiotherapy in Patients With Esophageal Adenocarcinoma | <https://clinicaltrials.gov/study/NCT02037048> |
|  | NCT02264678 | Ascending Doses of Ceralasertib in Combination With Chemotherapy and/or Novel Anti Cancer Agents | <https://clinicaltrials.gov/study/NCT02264678> |
| GASTRIC CANCER – Liquid Biopsy | NCT05029869 | Monitoring Minimal Residual Disease in Gastric Cancer by Liquid Biopsy Study Description | <https://clinicaltrials.gov/study/NCT05029869> |
|  | NCT06940778 | LIQUID BIOPSY FOCUSING ON CIRCULATING TUMOR CELLS AND CIRCULATING TUMOR DNA AS PRECISION MEDICINE IN GASTROINTESTINAL TUMORS | <https://clinicaltrials.gov/study/NCT06940778> |
|  | NCT06877910 | Deciphering the Molecular Traits of Non-canonical Responders to Advance Personalized Therapy in Gastric Cancer | <https://clinicaltrials.gov/study/NCT06877910> |
|  | NCT03835663 | The Bacterial Composition of the Stomach in Reflux Disease | <https://clinicaltrials.gov/study/NCT03835663> |
|  | NCT04817826 | TremelImumab aNd Durvalumab For the Non-operatIve Management (NOM) of MSI-high Resectable GC/GEJC. | <https://clinicaltrials.gov/study/NCT04817826> |
|  | NCT02381561 | Ropidoxuridine in Treating Patients With Advanced Gastrointestinal Cancer Undergoing Radiation Therapy | <https://clinicaltrials.gov/study/NCT02381561> |
| CHOLANGIOCARCINOMA - ctDNA | NCT06313203 | HAI-Floxuridine, or SIRT, Combined with Gemox for Patients with Intra-Hepatic Cholangiocarcinoma Not Amenable to Resection (TOMCAT) | <https://clinicaltrials.gov/study/NCT06313203> |
|  | NCT06638931 | Agnostic Therapy in Rare Solid Tumors | <https://clinicaltrials.gov/study/NCT06638931> |
|  | NCT05564403 | Study of Chemotherapy, With or Without Binimetinib in Advanced Biliary Tract Cancers in 2nd Line Setting (A ComboMATCH Treatment Trial) | <https://clinicaltrials.gov/study/NCT05564403> |
|  | NCT04526106 | REFOCUS: A First-in-Human Study of Highly Selective FGFR2 Inhibitor, RLY-4008, in Patients With ICC and Other Advanced Solid Tumors | <https://clinicaltrials.gov/study/NCT04526106> |
| COLORECTAL CANCER - ctDNA | NCT06143644 | Predictive Value of Postoperative Circulating Tumor DNA Monitoring for Colorectal Cancer Recurrence | <https://clinicaltrials.gov/study/NCT06143644> |
|  | NCT02842203 | Use of ctDNA for Monitoring of Stage III Colorectal Cancer | <https://clinicaltrials.gov/study/NCT02842203> |
|  | NCT04726800 | Circulating Tumour DNA (ctDNA) as a Prognostic and Predictive Marker in Colorectal Cancer - a Pilot Study | <https://clinicaltrials.gov/study/NCT04726800> |
|  | NCT03637686 | IMPROVE: Circulating Tumor DNA Analysis to Optimize Treatment for Patients With Colorectal Cancer | <https://clinicaltrials.gov/study/NCT03637686> |
|  | NCT05558436 | Comparison of Diagnostic Sensitivity Between ctDNA Methylation and CEA in Colorectal Cancer | <https://clinicaltrials.gov/study/NCT05558436> |
|  | NCT04264702 | BESPOKE Study of ctDNA Guided Therapy in Colorectal Cancer | <https://clinicaltrials.gov/study/NCT04264702> |
|  | NCT04084249 | ctDNA-guided Surveillance for Stage III CRC, a Randomized Intervention Trial | <https://clinicaltrials.gov/study/NCT04084249> |
|  | NCT03844620 | Circulating Cell-Free Tumor DNA Testing in Guiding Treatment for Patients With Advanced or Metastatic Colorectal Cancer | <https://clinicaltrials.gov/study/NCT03844620> |
|  | NCT05444491 | Application of Polygenic Methylation Markers in Postoperative Recurrence Monitoring of Colorectal Cancer | <https://clinicaltrials.gov/study/NCT05444491> |
|  | NCT05210283 | CORRECT Study of Minimal Residual Disease Detection in Colorectal Cancer | <https://clinicaltrials.gov/study/NCT05210283> |
|  | NCT06398743 | First CORRECT Study of Minimal Residual Disease (MRD) Detection in Colorectal Cancer | <https://clinicaltrials.gov/study/NCT06398743> |
|  | NCT06040632 | IMPROVE-pT1: Accurate Allocation of Completion Resection in Early Colorectal Cancer | <https://clinicaltrials.gov/study/NCT06040632> |
|  | NCT04813627 | Epidemiological Study to Monitor Study Participants With Resected Stage II (High Risk) or Stage III Colorectal Cancer for Circulating Tumor DNA Before, During and After Their Treatment With Adjuvant Chemotherapy | <https://clinicaltrials.gov/study/NCT04813627> |
|  | NCT06023004 | The Diverticulitis Study | <https://clinicaltrials.gov/study/NCT06023004> |
|  | NCT04259944 | Post-surgical Liquid Biopsy-guided Treatment of Stage III and High-risk Stage II Colon Cancer Patients: the PEGASUS Trial | <https://clinicaltrials.gov/study/NCT04259944> |
|  | NCT05674422 | GEMCAD-REVEAL STUDY - Circulating Tumor DNA as a Predictor of Relapse in Patients With Locally Advanced Rectal Cancer. | <https://clinicaltrials.gov/study/NCT05674422> |
|  | NCT05219734 | MRD Assay Evaluates Recurrence and Response Via a Tumor Informed Assessment | <https://clinicaltrials.gov/study/NCT05219734> |
|  | NCT04589468 | Researching the Effect of Exercise on Cancer | <https://clinicaltrials.gov/study/NCT04589468> |
|  | NCT03223779 | Study of TAS-102 Plus Radiation Therapy for the Treatment of the Liver in Patients With Hepatic Metastases From Colorectal Cancer | <https://clinicaltrials.gov/study/NCT03223779> |
|  | NCT04068103 | Circulating Tumor DNA Testing in Predicting Treatment for Patients With Stage IIA Colon Cancer After Surgery | <https://clinicaltrials.gov/study/NCT04068103> |
|  | NCT05710406 | Testing the Use of BRAF-Targeted Therapy After Surgery and Usual Chemotherapy for BRAF-Mutated Colon Cancer | <https://clinicaltrials.gov/study/NCT05710406> |
|  | NCT03776591 | Open D3 Right Hemicolectomy Compared to Laparoscopic CME for Right Sided Colon Cancer | <https://clinicaltrials.gov/study/NCT03776591> |
|  | NCT06940778 | LIQUID BIOPSY FOCUSING ON CIRCULATING TUMOR CELLS AND CIRCULATING TUMOR DNA AS PRECISION MEDICINE IN GASTROINTESTINAL TUMORS | <https://clinicaltrials.gov/study/NCT06940778> |
|  | NCT05708599 | A Study to Compare Tissue and Liquid Biopsies in People With Different Types of Cancer | <https://clinicaltrials.gov/study/NCT05708599> |
|  | NCT05726864 | A Study of ELI-002 7P in Subjects With KRAS/NRAS Mutated Solid Tumors | <https://clinicaltrials.gov/study/NCT05726864> |
|  | NCT05036109 | DAILY: Vitamin D, Aspirin, ExercIse, Low Saturated Fat Foods StudY in Colorectal Cancer Patients With Minimal Residual Disease | <https://clinicaltrials.gov/study/NCT05036109> |
|  | NCT05040568 | A PHASE IB STUDY OF IMMUNOTHERAPY WITH EX VIVO PRE-ACTIVATED AND EXPANDED CB-NK CELLS IN COMBINATION WITH CETUXIMAB, IN COLORECTAL CANCER PATIENTS WITH MINIMAL RESIDUAL DISEASE (MRD) | <https://clinicaltrials.gov/study/NCT05040568> |
|  | NCT04607421 | A Study of Encorafenib Plus Cetuximab With or Without Chemotherapy in People With Previously Untreated Metastatic Colorectal Cancer | <https://clinicaltrials.gov/study/NCT04607421> |
|  | NCT05570279 | PLAN-A Data- Bio- and Plan- Bank Collection for SCCA in Denmark | <https://clinicaltrials.gov/study/NCT05570279> |
|  | NCT05141721 | A Study of a Patient-Specific Neoantigen Vaccine in Combination With Immune Checkpoint Blockade for Patients With Metastatic Colorectal Cancer | <https://clinicaltrials.gov/study/NCT05141721> |
|  | NCT03635021 | Study to Evaluate the Efficacy of FOLFOX + Panitumumab Followed by FOLFIRI + Bevacizumab (Sequence 1) Versus FOLFOX + Bevacizumab Followed by FOLFIRI + Panitumumab (Sequence 2) in Untreated Patients With Wild-type RAS Metastatic, Primary Left-sided, Unresectable Colorectal Cancer | <https://clinicaltrials.gov/study/NCT03635021> |
|  | NCT04751773 | Postoperative Exercise Training in Patients With Colorectal Liver Metastases Undergoing Surgery (ELMA) | <https://clinicaltrials.gov/study/NCT04751773> |
|  | NCT06638931 | Agnostic Therapy in Rare Solid Tumors | <https://clinicaltrials.gov/study/NCT06638931> |
|  | NCT06106308 | Study of Onvansertib in Combination With FOLFIRI and Bevacizumab or FOLFOX and Bevacizumab Versus FOLFIRI and Bevacizumab or FOLFOX and Bevacizumab for First-Line Treatment of Metastatic Colorectal Cancer in Adult Participants With a KRAS or NRAS Mutation | <https://clinicaltrials.gov/study/NCT06106308> |
|  | NCT04046445 | Phase 1b Study to Evaluate ATP128, VSV-GP128 and BI 754091, in Patients With Stage IV Colorectal Cancer | <https://clinicaltrials.gov/study/NCT04046445> |
|  | NCT04719988 | Anti-PD-1 and mDCF Followed by Chemoradiotherapy in Patients With Stage III Squamous Cell Anal Carcinoma. | <https://clinicaltrials.gov/study/NCT04719988> |
|  | NCT04245865 | Tocotrienol and Bevacizumab in Metastatic Colorectal Cancer | <https://clinicaltrials.gov/study/NCT04245865> |
|  | NCT03975491 | The Exercise And Colorectal Cancer Treatment Trial | <https://clinicaltrials.gov/study/NCT03975491> |
| COLORECTAL CANCER - CTCs | NCT05818865 | "Principle Test" for Isolation and Characterization of Circulating Cancer Cells (CTC)-CXCR4+. | <https://clinicaltrials.gov/study/NCT05818865> |
|  | NCT05109130 | Change of Circulating Tumor Cells During Laparoscopic or Transanal Endoscopic Surgery for Rectal Cancer. | <https://clinicaltrials.gov/study/NCT05109130> |
|  | NCT03776591 | Open D3 Right Hemicolectomy Compared to Laparoscopic CME for Right Sided Colon Cancer | <https://clinicaltrials.gov/study/NCT03776591> |
|  | NCT06940778 | LIQUID BIOPSY FOCUSING ON CIRCULATING TUMOR CELLS AND CIRCULATING TUMOR DNA AS PRECISION MEDICINE IN GASTROINTESTINAL TUMORS | <https://clinicaltrials.gov/study/NCT06940778> |
|  | NCT05524012 | Longitudinal Multimodal Response Assessment During Neoadjuvant Treatment of Rectal Cancer | <https://clinicaltrials.gov/study/NCT05524012> |
|  | NCT02874885 | Circulating Tumor Cells in Patients With Locally Advanced Rectal Cancer | <https://clinicaltrials.gov/study/NCT02874885> |
|  | NCT04224779 | Liquid Biopsies and IMAging for Improved Cancer Care | <https://clinicaltrials.gov/study/NCT04224779> |
| COLORECTAL CANCER - EVs | NCT04852653 | A Prospective Feasibility Study Evaluating Extracellular Vesicles Obtained by Liquid Biopsy for Neoadjuvant Treatment Response Assessment in Rectal Cancer | <https://clinicaltrials.gov/study/NCT04852653> |
|  | NCT06730035 | PRediction of Outcomes and PERsonalized Radiotherapy by Biomarkers and Functional Imaging | <https://clinicaltrials.gov/study/NCT06730035> |
| COLORECTAL CANCER – Liquid Biopsy | NCT04852653 | A Prospective Feasibility Study Evaluating Extracellular Vesicles Obtained by Liquid Biopsy for Neoadjuvant Treatment Response Assessment in Rectal Cancer | <https://clinicaltrials.gov/study/NCT04852653> |
|  | NCT05708599 | A Study to Compare Tissue and Liquid Biopsies in People With Different Types of Cancer | <https://clinicaltrials.gov/study/NCT05708599> |
|  | NCT04224779 | Liquid Biopsies and IMAging for Improved Cancer Care | <https://clinicaltrials.gov/study/NCT04224779> |
|  | NCT04259944 | Post-surgical Liquid Biopsy-guided Treatment of Stage III and High-risk Stage II Colon Cancer Patients: the PEGASUS Trial | <https://clinicaltrials.gov/study/NCT04259944> |
|  | NCT06940778 | LIQUID BIOPSY FOCUSING ON CIRCULATING TUMOR CELLS AND CIRCULATING TUMOR DNA AS PRECISION MEDICINE IN GASTROINTESTINAL TUMORS | <https://clinicaltrials.gov/study/NCT06940778> |
|  | NCT05674422 | GEMCAD-REVEAL STUDY - Circulating Tumor DNA as a Predictor of Relapse in Patients With Locally Advanced Rectal Cancer. | <https://clinicaltrials.gov/study/NCT05674422> |
|  | NCT05524012 | Longitudinal Multimodal Response Assessment During Neoadjuvant Treatment of Rectal Cancer | <https://clinicaltrials.gov/study/NCT05524012> |
|  | NCT04034459 | FOLFOXIRI Plus Cetuximab vs. FOLFOXIRI Plus Bevacizumab 1st-line in BRAF-mutated mCRC | <https://clinicaltrials.gov/study/NCT04034459> |
|  | NCT00625066 | Biological, Genetic, and Lifestyle Risk Factors for Developing Colorectal Adenomas or Polyps in Participants Undergoing Colonoscopy | <https://clinicaltrials.gov/study/NCT00625066> |
|  | NCT05312398 | CAPRI 2 GOIM Study: Investigate the Efficacy and Safety of a Bio-marker Driven Cetuximab-based Treatment Regimen | <https://clinicaltrials.gov/study/NCT05312398> |
|  | NCT02381561 | Ropidoxuridine in Treating Patients With Advanced Gastrointestinal Cancer Undergoing Radiation Therapy | <https://clinicaltrials.gov/study/NCT02381561> |
|  | NCT04261972 | Cell-free DNA in Hereditary And High-Risk Malignancies | <https://clinicaltrials.gov/study/NCT04261972> |
|  | NCT04046445 | Phase 1b Study to Evaluate ATP128, VSV-GP128 and BI 754091, in Patients With Stage IV Colorectal Cancer | <https://clinicaltrials.gov/study/NCT04046445> |
|  | NCT03776591 | Open D3 Right Hemicolectomy Compared to Laparoscopic CME for Right Sided Colon Cancer | <https://clinicaltrials.gov/study/NCT03776591> |
|  | NCT04246684 | Short RT Versus RCT, Followed by Chemo.and Organ Preservation for Interm and High-risk Rectal Cancer Patients | <https://clinicaltrials.gov/study/NCT04246684> |
| PANCREATIC CANCER - ctDNA | NCT07122466 | Early Treatment ctDNA Dynamics to Predict Response to Chemotherapy | <https://clinicaltrials.gov/study/NCT07122466> |
|  | NCT04241367 | Verification of Predictive Biomarkers for Pancreatic Cancer Treatment Using Multicenter Liquid Biopsy | <https://clinicaltrials.gov/study/NCT04241367> |
|  | NCT04246203 | Prognostic Role of Circulating Tumor DNA in Resectable Pancreatic Cancer | <https://clinicaltrials.gov/study/NCT04246203> |
|  | NCT06940778 | LIQUID BIOPSY FOCUSING ON CIRCULATING TUMOR CELLS AND CIRCULATING TUMOR DNA AS PRECISION MEDICINE IN GASTROINTESTINAL TUMORS | <https://clinicaltrials.gov/study/NCT06940778> |
|  | NCT05708599 | A Study to Compare Tissue and Liquid Biopsies in People With Different Types of Cancer | <https://clinicaltrials.gov/study/NCT05708599> |
|  | NCT05638698 | Tg01 Vaccine / Qs-21 Stimulon™ With Or Without Balstilimab As Maintenance Therapy Following Adjuvant Chemotherapy In Patients With Resected Pancreatic Cancer | <https://clinicaltrials.gov/study/NCT05638698> |
|  | NCT05726864 | A Study of ELI-002 7P in Subjects With KRAS/NRAS Mutated Solid Tumors | <https://clinicaltrials.gov/study/NCT05726864> |
|  | NCT05068752 | Phase II Trial of Vemurafenib and Sorafenib in Pancreatic Cancer | <https://clinicaltrials.gov/study/NCT05068752> |
|  | NCT04672005 | Modified FOLFIRINOX Alternated With Biweekly Gemcitabine Plus Nab-Paclitaxel Untreated Pancreatic Cancer | <https://clinicaltrials.gov/study/NCT04672005> |
|  | NCT04940286 | Gemcitabine, Nab-paclitaxel, Durvalumab, and Oleclumab Before Surgery for the Treatment of in Resectable/Borderline Resectable Primary Pancreatic Cancer | <https://clinicaltrials.gov/study/NCT04940286> |
|  | NCT05554367 | Palbociclib and Binimetinib in RAS-Mutant Cancers, A ComboMATCH Treatment Trial | <https://clinicaltrials.gov/study/NCT05554367> |
|  | NCT02498613 | A Phase 2 Study of Cediranib in Combination With Olaparib in Advanced Solid Tumors | <https://clinicaltrials.gov/study/NCT02498613> |
| PANCREATIC CANCER - CTCs | NCT06940778 | LIQUID BIOPSY FOCUSING ON CIRCULATING TUMOR CELLS AND CIRCULATING TUMOR DNA AS PRECISION MEDICINE IN GASTROINTESTINAL TUMORS | <https://clinicaltrials.gov/study/NCT06940778> |
|  | NCT05085548 | ProAgio in Previously Treated Advanced Pancreatic Cancer and Other Solid Tumor Malignancies | <https://clinicaltrials.gov/study/NCT05085548> |
| PANCREATIC CANCER – Liquid Biopsy | NCT04246203 | Prognostic Role of Circulating Tumor DNA in Resectable Pancreatic Cancer | <https://clinicaltrials.gov/study/NCT04246203> |
|  | NCT04241367 | Verification of Predictive Biomarkers for Pancreatic Cancer Treatment Using Multicenter Liquid Biopsy | <https://clinicaltrials.gov/study/NCT04241367> |
|  | NCT06940778 | LIQUID BIOPSY FOCUSING ON CIRCULATING TUMOR CELLS AND CIRCULATING TUMOR DNA AS PRECISION MEDICINE IN GASTROINTESTINAL TUMORS | <https://clinicaltrials.gov/study/NCT06940778> |
|  | NCT05708599 | A Study to Compare Tissue and Liquid Biopsies in People With Different Types of Cancer | <https://clinicaltrials.gov/study/NCT05708599> |
|  | NCT03851133 | Florida Pancreas Collaborative Next Generation Biobank | <https://clinicaltrials.gov/study/NCT03851133> |
|  | NCT06334458 | Epigenomic and Machine Learning Models to Predict Pancreatic Cancer | <https://clinicaltrials.gov/study/NCT06334458> |
|  | NCT03305146 | Feasibility of Molecular Biology in Pancreatic Cyst Tumors | <https://clinicaltrials.gov/study/NCT03305146> |
|  | NCT02381561 | Ropidoxuridine in Treating Patients With Advanced Gastrointestinal Cancer Undergoing Radiation Therapy | <https://clinicaltrials.gov/study/NCT02381561> |
| LIVER CANCER - ctDNA | NCT03839706 | Relationship Between 18FDG PET/MRI Patterns and ctDNA to Predict HCC Recurrence After Liver Transplantation | <https://clinicaltrials.gov/study/NCT03839706> |
|  | NCT03223779 | Study of TAS-102 Plus Radiation Therapy for the Treatment of the Liver in Patients With Hepatic Metastases From Colorectal Cancer | <https://clinicaltrials.gov/study/NCT03223779> |
|  | NCT04751773 | Postoperative Exercise Training in Patients With Colorectal Liver Metastases Undergoing Surgery (ELMA) | <https://clinicaltrials.gov/study/NCT04751773> |
|  | NCT04046445 | Phase 1b Study to Evaluate ATP128, VSV-GP128 and BI 754091, in Patients With Stage IV Colorectal Cancer | <https://clinicaltrials.gov/study/NCT04046445> |
|  | NCT02180867 | Radiation Therapy With or Without Combination Chemotherapy or Pazopanib Before Surgery in Treating Patients With Newly Diagnosed Non-rhabdomyosarcoma Soft Tissue Sarcomas That Can Be Removed by Surgery | <https://clinicaltrials.gov/study/NCT02180867> |
|  | NCT04196465 | Phase II Study of Neoadjuvant Immune Checkpoint Inhibitor in Patients With Resectable Gastrointestinal Cancers | <https://clinicaltrials.gov/study/NCT04196465> |
| LIVER CANCER - CTCs | NCT02364557 | Testing Whether Treating Breast Cancer Metastases With Surgery or High-Dose Radiation Improves Survival | <https://clinicaltrials.gov/study/NCT02364557> |
| LIVER CANCER – Liquid Biopsy | NCT06703853 | Identifying Tissue-of-origin in Transplant Patients and Patients with Malignancies | <https://clinicaltrials.gov/study/NCT06703853> |
|  | NCT02261415 | The HeLiX (Hemorrhage During Liver Resection: traneXamic Acid) Trial | <https://clinicaltrials.gov/study/NCT02261415> |
|  | NCT02381561 | Ropidoxuridine in Treating Patients With Advanced Gastrointestinal Cancer Undergoing Radiation Therapy | <https://clinicaltrials.gov/study/NCT02381561> |
|  | NCT03278925 | Defined Green Tea Catechin Extract in Preventing Liver Cancer in Participants With Cirrhosis | <https://clinicaltrials.gov/study/NCT03278925> |
|  | NCT04046445 | Phase 1b Study to Evaluate ATP128, VSV-GP128 and BI 754091, in Patients With Stage IV Colorectal Cancer | <https://clinicaltrials.gov/study/NCT04046445> |
